# A review of nonstandardized applicators digitization in Nucletron™ HDR procedures

**DOI:** 10.1002/acm2.12156

**Published:** 2017-08-30

**Authors:** Kelin Wang, Michele S. Ferenci, Alberto de la Zerda, Kyle R. Padgett, Elizabeth L. Bossart, Ming Chao, Hua Shao, Mutain Zhang

**Affiliations:** ^1^ Department of Radiology Radiation Oncology Penn State Milton S. Hershey Medical Center Hershey PA USA; ^2^ Department of Radiation Oncology Sylvester Comprehensive Cancer Center University of Miami Miami FL USA; ^3^ Department of Radiation Oncology Icahn School of Medicine at Mont Sinai New York NY USA; ^4^ 21st Century Oncology Kendall Oncology Center Miami FL USA; ^5^ Department of Radiation Oncology University of Nebraska Medical Center Omaha NE USA

**Keywords:** digitization, FDP, non‐CT‐compatible, nonstandardized applicator, TCL

## Abstract

The major errors in HDR procedures were failures to enter the correct treatment distance, which could be caused by either entering wrong transmission lengths or imprecisely digitizing the dwelling positions. Most of those errors were not easily avoidable by enhancing the HDR management level because they were caused by implementations of nonstandardized applicators utilizing transmission tubes of different lengths in standard HDR procedures. We performed this comprehensive study to include all possible situations with different nonstandardized applicators that frequently occurred in HDR procedures, provide corresponding situations with standard applicator as comparisons, list all possible errors and in planning, clarify the confusions in offsets setting, and provide mathematical and quantitative solutions for each given scenarios. Training on HDR procedures with nonstandardized applicators are normally not included in most residential program for medical physics, thus this study could be meaningful in both clinical and educational purpose. At precision of 1 mm, our study could be used as the essential and practical reference for finding the correct treatment length as well as locating the accurate dwelling positions in any HDR procedure with nonstandardized applicators.

## 1 INTRODUCTION

Brachytherapy plays an indispensable role in radiation oncology. In recent years, high‐dose rate (HDR) brachytherapy procedures increasingly replaced low‐dose rate brachytherapy, for example, in the treatment of cervix cancers.^1^, ^2^, ^3^, ^4^, ^5^, ^6^, ^7^, ^8^, ^9^ However, the rapid delivery of HDR allows for little room to remedy errors that occur from time to time.^10^ The most common error in administering the HDR brachytherapy procedure is failure to enter the correct treatment distance,^11^ caused by either incorrect channel length or imprecisely digitized dwell positions. It is possible that errors could be reduced by the enhancement of quality assurance (QA) management, but some errors are more likely to result from the drawbacks of nonstandard applicators. These errors are relatively difficult to avoid.

Old fashioned HDR brachytherapy procedures were based on orthogonal radiographs, manual digitizer and tables for dwelling time with decay corrections.^12^ HDR applicators evolved in sync with the evolution of HDR brachytherapy, of course, many of these HDR applicators were novel developments in their time, such as vaginal cylinders of assemblability design, and the Miami applicators for multiple clinical uses.^13^ After years of clinical implementation, however, those early ideas were gradually adopted by brachytherapy vendors that started to market new, more powerful, and standardized applicators to meet the increasing use of HDR brachytherapy for different anatomical sites. Thus, contemporary HDR applicators tend to be standardized in several essential aspects. First, many applicators are compatible with radiograph or computed tomography (CT); i.e., when metallic markers are inserted into the applicators, they are visible in radiographs as well as in CT slices. Although applicators compatible to both CT and magnetic resonance (MR) have been developed for image‐guided HDR brachytherapy, as recommended by GEC‐ESTRO,^14^ HDR planning is more frequently CT‐based than MR‐based. Second, in rigid applicators for example, gynecology (GYN) Tandem & Ovoid applicators by Nucletron™ (an Elekta Company), transfer tubes are applicator‐specific and not exchangeable, and the first dwell position (FDP, also referred to as reference distance) of the radioactive source is standardized. Third, when flexible applicators (such as MammoSite of Hologic, Inc.) are implanted, total channel lengths (TCLs) are typically shorter than those of the rigid applicators and must be measured for each applicator or channel.

Nevertheless, these nonstandardized HDR applicators are still being used in many cancer centers nationwide. The word “nonstandardized” hereafter refers to applicators that are either non‐CT compatible, or that do not have applicator‐specific transfer tubes, or both. Compared to the new and sophisticated HDR applicators, those “old‐fashioned” ones were mostly customized for treatment sites with less concern for user convenience, and hence bring about drawbacks in multiple aspects. The greatest advantage of old‐fashioned applicators, however, is that for the most part they were designed to be compatible with standardized transfer tubes. Therefore, there is no need to purchase extra transfer tubes particularly designed for them.

It is required that full calibration of an HDR unit should include determination of source positioning accuracy to within ±1 mm.^15^ The typical verification or QA methods for HDR brachytherapy source dwell positions were proposed so that radiographic films could be used in direct contact with applicators.^16^ According to the recommendations of the AAPM TG‐59 report,^17^ in recent years, revised radiographic methods for dwell position measurement were reported for specific applicators^17^; other novel methods, such as using fluorescent screens were also proposed for dwell position verification.^18^ With the recent development of image‐guided radiotherapy, new instrumentation as well as planning tips were proposed for precise digitization of applicators in rapid HDR procedure workflows.^19^, ^20^ All those endeavors were essentially based on regular applicators implemented in HDR brachytherapy procedures. Difficulties or issues in digitizing nonstandardized applicators in prevalent procedures are always major causes for treatment errors.

Nonstandardized HDR applicators are neither CT nor radiograph compatible, and therefore markers are invisible in the images even if they were inserted to the applicators before the CT scan or radiography. Though some nonstandardized applicators meet the second or third aspect of standardization mentioned above, there are always many exceptions. For instance, when an older Miami applicator^13^ is implanted for an HDR brachytherapy on GYN cervix cases, applicator‐specific transfer tubes for vaginal cylinder applicators are used for all five channels (one channel for the tandem and four for the ovoids respectively). TCLs of the ovoids are much shorter than that of the tandem, one obvious drawback. In Table 1, scenarios for implementing nonstandardized applicators are listed, compared to implementing standardized applicators.

**Table 1:** Comparisons of standardized to nonstandardized applicators in combination with applicator‐specific or general transfer tubes, based on Nucletron™ products

Applicator/Tubes	Standardized rigid applicators	Standardized flexible applicators	Nonstandardized applicators
Applicator‐specific transfer tubes	Applicable yes	Not applicable	Applicable yes
	Reference distance = 1500 mm		Reference distance ≤1500 mm
	CT compatible (markers visible)		Not CT compatible (markers invisible)
Nonspecific transfer tubes	Not applicable	Applicable Yes	Applicable Yes
		Reference distance ≤1500 mm	Reference distance ≤1500 mm
		CT compatible (markers visible)	Not CT compatible (markers invisible)

As the major advantage for nonstandardized applicator designs, compatibility to existing applicator‐specific transfer tubes is also a costly tradeoff because these can cause errors in digitization. Once an applicator is commissioned, it could be used properly for a long time regardless of standardization. However, using nonstandardized HDR applicators has been challenging to medical physicists, making it necessary and crucial to have comprehensive preparations prior to the procedure. According to our experience, in most CAMPEP residency programs, medical physicist residents are formally trained to perform HDR procedures only under standardized conditions. Experienced medical physicists who rarely have exposure to customized applicators would also expect an inevitable learning curve in their practice. Here, we address the major issues in commissioning nonstandardized HDR applicators, and provide a comprehensive guideline on digitization for all conditions with nonstandardized applicators. For convenience and consistency, standardized Nucletron applicators and transfer tubes are used as references.

## 2 GENERAL PRINCIPLES

### 2.A Total channel length, reference length, and the first dwell position

**Figure 1:**
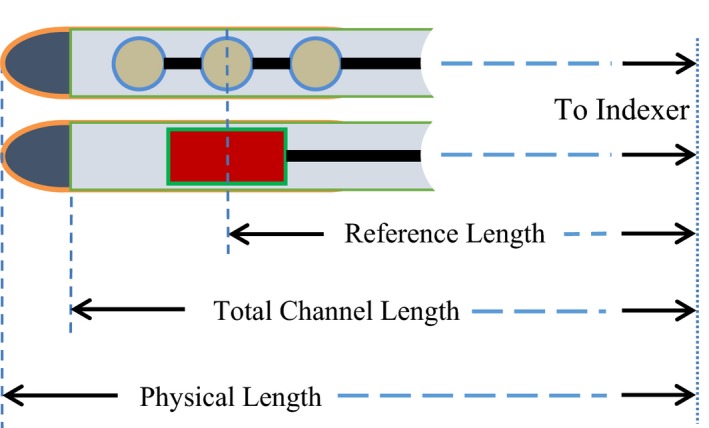
The physical length, total channel length, and reference length of an HDR applicator when connected through a transfer tube to the remote afterloader.

Several important terms should be defined for an HDR applicator connected to a transfer tube. The physical length of the applicator is counted from the remote afterloader indexer to the tip of the applicator, as determined by either mechanical or radiographic means; the total channel length (TCL), also referred to as total internal length, is the distance between the indexer to the inner end of the applicator channel; and the reference length is the distance between the indexer and the center of the source at the first dwell position (FDP). The reference length is numerically equal to the FDP, which is identical to the position indicated by the central bead of the metallic marker in radiographic film. These terms are illustrated in Fig. 1.

In typical HDR procedures, either tip‐end or connector‐end digitization could be performed, depending on user's preference. The most updated treatment planning system (TPS) uses tip‐end digitization by default, and therefore here we will concentrate on that method. In tip‐end digitization, distance is measured from the remote afterloader indexer, and thus all lengths and dwell positions have positive values. For the purpose of applicator digitization, it is often important to measure the distance from the applicator tip to the FDP, which is thereafter termed the offset; the offset should have a negative value because the positive direction points toward the indexer.

The HDR vendor provides a simulator wire with built‐in x‐ray markers. The end portion of the wire consists of three metallic beads, shown as three consecutive bright spots on CT slices. The central spot is defined as the tip‐end marker position. This is not the physical end of the applicator channel, even if the front marker bead touches the inner end. The end‐to‐end length of the three metallic beads is exactly 8 mm. If the front marker bead touches the channel end (the zero‐gap scenario), there will be a 4 mm distance between the tip‐end marker position and the channel end, and in that case the tip‐end marker position is digitized as the FDP of the ^192^Ir source. In this scenario, to maintain a necessary safety margin, there will be a 2 mm gap between the applicator channel end and the tip of the active source when located at the FDP.

Nonstandardized applicators, however, are manufactured such that they have slightly longer TCLs than standard applicators; these usually satisfy the zero‐gap scenario. However, if the x‐ray markers are extended in the nonstandardized applicators by the same distance as in standardized applicators, the front marker will see a distance of up to a few millimeters between front marker and the channel end (nonzero‐gap scenario). This common feature in nonstandardized applicators makes their digitization different (at least in principle) from that of standardized applicators.

### 2.B Use of rulers supplied by vendors

**Figure 2:**
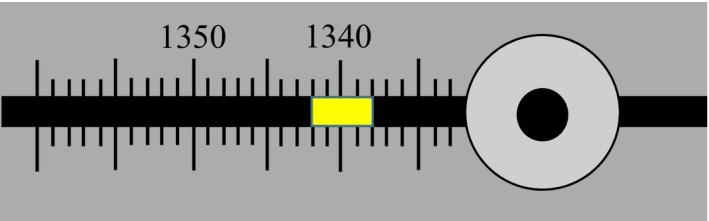
The position indicator (yellow piece) of a SPS, with its center located at 1340 mm. If the dummy wire of the simulator reaches the end of applicator, the indicator forehead shows the TCL (1342 mm), whereas its tail‐end indicates the reference distance or FDP (1338 mm).

Even if an HDR catheter and the x‐ray markers can be digitized in CT image or radiograph, the user must perform measurements to determine or verify the lengths defined in the previous section. These measurements have to be accomplished using tools supplied by vendors; for example, the source position simulator (SPS) set for microSelectron (Fig. 2) and the source position check ruler (SPCR) by Nucletron. SPS is for predelivery length measurements. SPCR is more often for real delivery length QA. While using SPCR, the measured length should be the central position of the source as indicated in the ruler. However, while using SPS, the measured lengths depend on situations of applicators and transfer tubes, as discussed below.

For standardized rigid HDR applicators, the first step of digitization is completed beforehand: the FDP is always 1500 mm as defaulted in TPS. For standardized flexible HDR applicators, the TCL varies channel by channel and case by case, so does the FDP. Thus, the TCL should always be measured with the SPS (Fig. 2) at least once prior to treatment.

**Figure 3:**
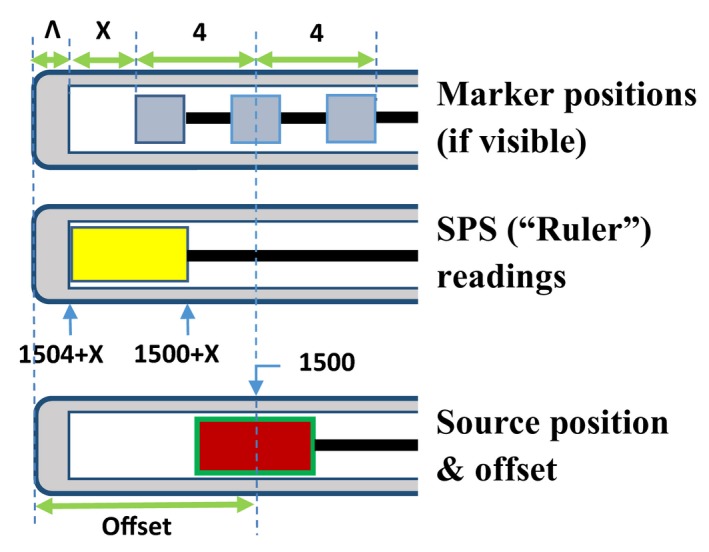
The relative positions of x‐ray markers, SPS, and ^192^Ir source in an applicator that has a reference distance of 1500 mm. Λ is the tip‐end thickness, and *x* is the gap between the applicator end and the front marker. Please note that markers might be invisible in a nonstandardized applicator.

In general, the reference distance of an applicator might not be exactly 4 mm from the channel end (nonzero‐gap scenario). To take into account all possible scenarios, Fig. 3 shows the general situation of digitizing an HDR channel, but using a standardized applicator as an example. The offset of the FDP consists of the tip‐end wall thickness *Λ*, the gap *x* between inner end of applicator channel and the front marker, and half‐length (4 mm) of the x‐ray markers. The parameters are correlated with the follow equation:

(1) $$\begin{array}{cc} & FDP=1500\\ & offset=-(\Lambda +x+4)\\ & TCL=FDP+4+x \end{array}$$

**Figure 4:**
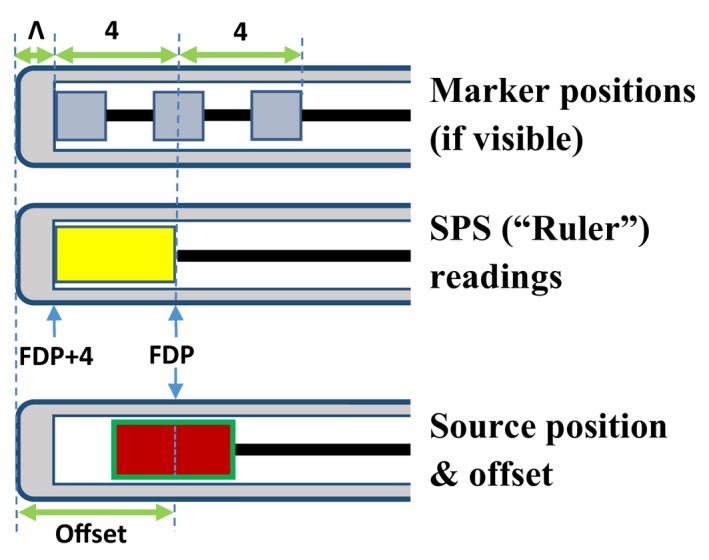
Relative positions of x‐ray markers, source position simulator, and active Ir‐192 source in a typical flexible applicator. The position of the central metallic mass is the position of the first dwell position (FDP) of the Ir‐192 source, whereas the center of the SPS dummy source is located at FDP+2 mm. Please note the FDP could be any arbitrary number less than 1500 mm.

The negative sign of offset means TCL is longer than the reference length, as required by the TPS. It should be noted that if *x* = 0*,* the equation reduces to the zero‐gap scenario, as shown later in Fig. 4. In fact, if the FDP is 1500 mm, it is rare that *x* is exactly zero. Furthermore, zero‐gap scenarios occur more commonly in the channels of flexible applicators where FDP is less than 1500 mm.

### 2.C Digitization of an HDR applicator

This subsection presents the principles of HDR applicator digitization that are common to both standardized and nonstandardized applicators (implementation of these principles is addressed in the next section). The first step of applicator digitization is to determine its total length, usually required by the TPS. The second step is to determine the tip‐end marker position or the FDP. Next, the user digitizes the remaining dwell positions towards the connector end to allow for sufficient treatment length. For nonstandardized HDR applicators, special care should be always taken in the first two steps regardless of the transfer tubes being used. It is critical to note that each step could present challenges due to nonstandardization. The third step is not generally applicable for nonstandardized applicators, since many of them are not radiograph or CT compatible, and thus reasonable estimations are typically introduced.

The TCL and FDP can always be measured using SPS and x‐ray markers respectively. The accuracy of SPS measurement can be verified using the source position check ruler, which has a 2‐cm position indicator that can be pushed along the ruler channel via the simulation wire or dummy cable installed on the SPS. The proximal end reading of the position indicator is the extended length of the simulation wire or the dummy cable. According to user manuals, the SPS and the check ruler have an accuracy of ±0.5 mm. Detailed methods of using the SPS and the check ruler are described in the user manuals.

## 3 PRACTICAL APPROACH

### 3.A Digitization of rigid applicators with applicator‐specific transfer tubes

Equation (1) holds true for HDR applicators implemented with applicator‐specific transfer tubes, whether or not they are standardized. For a CT‐compatible applicator, the tip‐end marker position can be located by digitizing the center of the three x‐ray markers; this position is the FDP of the ^192^Ir source in the zero‐gap scenario. More often than not, however, the gap between the tip‐end metallic bead and the channel end is nonzero (Fig. 3). If so, the SPS proximal end readout will be larger than the intended FDP. Thus, it is a big mistake to use an SPS‐based length measurement with the rigid applicators supplied by vendors. The SPS measurement of the TCL is for verifying applicator length during periodic QA.

Accurate measurement of the TCL is crucial for non‐standardized applicator digitization. Of all nonstandardized rigid applicators, vaginal cylinders with assemblability design are probably the simplest. A typical applicator set normally consists of a very thin metallic catheter with a series of cylindrical applicators in different diameters. Each cylindrical applicator part has a central hole through which the metallic catheter may penetrate. The cylindrical parts are made of water‐equivalent materials, with the metallic wall of the catheter quite thin to avoid excessive dose attenuation. No x‐ray markers are available for these applicators. As a major concern for user convenience, those nonstandardized vaginal cylinder applicators are compatible with the standard applicator‐specific transfer tubes for standard vaginal cylinder applicators. The FDP is presumably 1500 mm.

The major challenge to digitize this type of nonstandardized catheter is to find the FDP on the radiograph or a CT slice. Because no x‐ray markers can be used, both *x* and *Λ* (Fig. 3) must be determined, but the inner gap *x* can be easily determined with the SPS. For instance, if the SPS tail‐end reading is 1503 mm for an applicator of 1500 mm FDP, the inner gap *x* = 1503‐1500 = 3 (mm).

The catheter's metallic shell is constructed normally quite thin to minimize attenuation; thus *Λ* is typically within 1–2 mm, and could be measured with the SPS and autoradiograph. The procedures are to: (a) measure the channel length of the catheter with the SPS, (b) make a simple plan of only one dwelling point at the FDP; and (c) place a scaled film with the applicator channel and deliver the plan. The length between the center of the exposure spot and the outer end of applicator is the applicator offset in eq. (1), noting that offset is a negative number, thus

(2) Λ=−offset−x−4

### 3.B Digitization of flexible applicators with non‐applicator‐specific transfer tubes

Flexible applicators, whether or not standardized, are implemented by default with nonapplicator‐specific transfer tubes. These applicators are suitable for treating irregular or large treatment sites, such as sarcomas with interstitial implants, or skin melanomas with Nucletron™ Freiburg Flap Applicator Set. Standard catheters compatible with the connectors of nonapplicator‐specific transfer tubes are normally used, and are typically ~20 cm shorter than those with applicator‐specific transfer tubes.

The reference catheter supplied by the vendor plays the key role of defining the TCL for each catheter. All catheters are cut manually according to the zero‐marker position of the reference catheter. The distance between the end of channel and the center of reference zero‐marker position is around several millimeters, and accounts for the uncertainty of manually cut catheter lengths. In reality, the actual TCL of each catheter may vary slightly, and hence should be measured with an SPS after the catheter is cut.

Prior to the CT scan or radiography, x‐ray markers are fully inserted into each applicator channel until the tip of the markers reach the end of each given channel. As mentioned earlier, similar to standardized rigid applicators with applicator‐specific tubes, the tip‐end marker position can be easily digitized in a radiograph or CT slice. However, unlike that shown in Fig. 3, there is no extra space ahead of the front‐end metallic bead of the markers in each channel because the markers are fully inserted. In this situation, the gap *x* is zero and is essentially irrelevant in digitization of the flexible applicator. While measuring the TCL with the SPS, the forehead of the dummy source touches the catheter channel end, and its tail‐end is the exact central position of the 3 metallic beads, which is also the center of real ^192^Ir source at its first dwelling position, if a 2‐mm gap is chosen between the channel end and the tip of the active source.

Using a skin case as an example, with Freiburg Flap Applicator Set of 5 applicator channels, if the central position of the SPS position indicator is 1292 mm, the TCL and the FDP should be digitized as the tip‐end reading (1294 mm) and tail‐end reading (1290 mm) respectively. Figure 4 illustrates the relative positions of the x‐ray markers, the dummy source of SPS, and the real ^192^Ir source in this example.

Of course, there are exceptions. The endobronchial applicators (or other similar applicators) are simple catheters of 1500 mm in length, and thus no extra transfer tubes are needed. The digitization for those simple applicators is identical to the scenario of standardized transfer tubes with reusable applicators, for example, GYN vaginal cylinders.

### 3.C Digitization of non‐CT‐compatible applicators with shorter TCL

Nonstandardized applicators are mostly made of metallic materials, and indeed it is uncommon to see nonstandardized flexible applicators, though standardized flexible applicators are routinely implemented. Nonstandardized applicators were made compatible to not only applicator‐specific transfer tubes, but also nonapplicator‐specific transfer tubes, because compatibility depends only on the connector types. In case they are implemented with nonapplicator‐specific transfer tubes, as mentioned earlier, there is no reference catheter for zero‐marker position available, and the FDP needs to be measured with the SPS for each channel. It is not unusual to find that applicators are short in length but still implemented with applicator‐specific transfer tubes, so their FDPs are much shorter than 1500 mm. This could cause confusion, because the default FDP is always 1500 mm whenever an applicator‐specific transfer tube is used with a standardized applicator.

Given that in such an applicator the FDP is shorter than 1500 mm, the dwell positions of the radioactive source will be accordingly shorter. In the digitization, we assume the zero‐gap scenario for the dummy source when the SPS is used to measure the TCL and the FDP. To measure the tip‐end thickness Λ, the same procedures addressed in Section 3.A may be followed. The formula for determining the FDP and the offset is:

(3) $$\begin{array}{cc} & FDP=SPStail-endreading\\ & offset=-(\Lambda +4) \end{array}$$

For negligible applicator thickness (Λ = 0), the offset can be set to 4 mm. In most cases, the typical offsets are 4–6 mm. The digitization procedure is also similar to that shown in Fig. 4, except that the metallic markers are invisible in these non‐CT‐compatible applicators.

### 3.D Common errors in digitizing non‐CT‐compatible applicators

Two types of errors frequently occur in digitizing nonstandardized applicators with applicator‐specific transfer tubes; i.e., applicators are not CT compatible, with FDP presumably to be 1500 mm.

The first type of error (Type‐I) in digitization (also the most common one) is to directly digitize the physical tip of the catheter from the CT or the radiograph as the tip‐end marker position, using 1500 mm as the FDP as well as zero offset. Thus the inner gap *x*, the tip‐end thickness Λ, and the size of the tip‐end beads of the markers are ignored.

**Figure 5:**
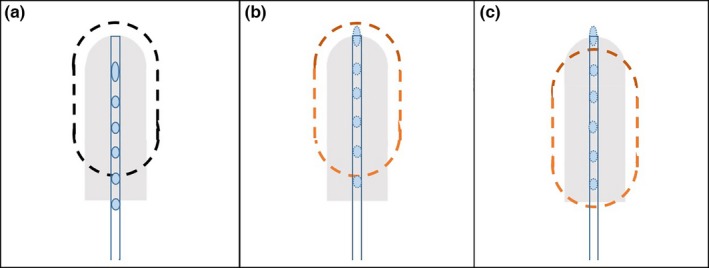
Type‐I errors for vaginal cylinder applicators. Panel A illustrates the situation with standardized applicator. Panel B illustrates the Type‐I errors with nonstandardized applicator: in TPS the planned IDL is similar to that in Panel A but with different dosimetry parameters. Panel C illustrates the delivered IDL if a Type‐I error occurred in digitization: the IDL is moved downstream compare to panel B.

For single channel applicators e.g., vagina cylinders, a Type‐I error moves the delivered isodose lines (IDLs) downstream compared to the IDLs seen at the TPS, as shown in Fig. 5. Panel A of Fig. 5 illustrates the correct IDL based on a standardized applicator with visible markers; panel B illustrates the wrong digitization (Type‐I error) and the planned IDL shown on the TPS. The treatment plans in panel A and B are different, although the IDLs are similar since they were for the same prescription. However, considering 1500 mm FDP, the delivered IDL is not as seen in the TPS (panel B of Fig. 5), but instead is of the same pattern but downstream by the length ignored in digitization, as shown in panel C. As discussed above, the exact downstream length is depicted in eq. (2).

**Figure 6:**
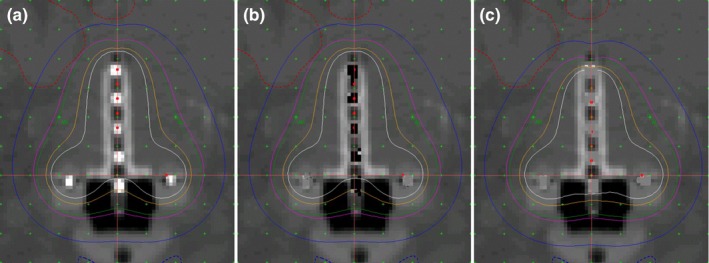
Type‐I errors for tandem & ring applicators. Panel A illustrates the situation with standardized applicator. Panel B illustrates the Type‐I errors in tandem with nonstandardized applicator: in TPS the planned IDL is similar to that in Panel A but with different dosimetry parameters. Panel C illustrates the delivered IDL, which was reshaped downstream from panel B.

For Tandem & Ring applicator for instance, digitization with Type‐I error at the tandem reshapes the IDLs, causing incorrect delivered doses in reference points A & points B, as shown in Fig. 6. Panel A of Fig. 6 illustrates the IDLs of a standard Tandem & Ring applicator, with dose normalized to reference points A. Panel B illustrates the planned IDLs in TPS for a nonstandardized Tandem & Ring applicator with wrong digitization (Type‐I error), though the IDLs look similar to that in panel A. However, considering 1500 mm FDP, the delivered IDLs are reshaped because the dose delivered in the tandem moved downstream, as shown in panel C.

Because the allowed uncertainty for HDR planning is 2 mm (AAPM TG‐40 21), Type‐I errors are much greater than can be tolerated. Of note, Type‐I errors can also occur when digitizing nonstandardized applicators with shorter channel lengths.

The second type of common error (Type‐II) in digitization is to ignore the inner gap *x* of eq. (2) when it is not zero, and directly implementing eq. (3). Since the other components of the offset are already taken into account, the difference in dose distributions caused by this type of error is solely dependent on the magnitude of the inner gap *x*. Similar to Type‐I errors, in this case the actual dose distribution should be of the same pattern, but instead is shifted downstream by the distance *x*. Panel B & C of Figs. 5 and 6 are still applicable to Type‐II errors, except that the downstream length is equivalent to inner gap *x*.

It is important to point out that neither Type‐I nor Type‐II errors will be identified by the remote afterloader system, because the source always travels as far as the FDP, and this is true even if the digitized spot is at a distance beyond it. This is also the reason that those errors could not be easily discovered, because delivery may go through successfully. If a positive offset rather than a negative offset is typed into the TPS, however, this error can be rejected by the delivery system, because in this situation the dummy wire will attempt to travel beyond the physical end of the catheter channel.

**Figure 7:**
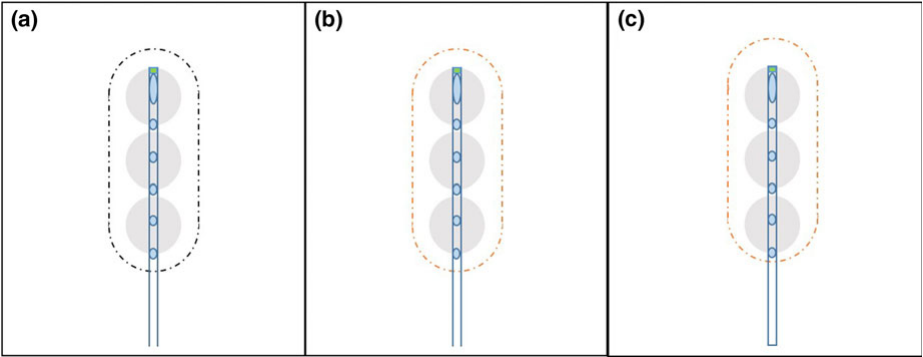
Type‐III errors. Panel A illustrates the correct digitization for SPS position indicator. Panel B illustrates the incorrect digitization (Type‐III error) for SPS position indicator, although in TPS the IDL in panel B is identical to that in Panel A. Panel C indicates the delivered IDL, which was moved 2 mm upstream compare to panel A.

Another common mistake (Type‐III) is to digitize the FDP by reading the central position of the SPS position indicator, and this mistake occurs frequently when flexible applicators are implemented. Because the SPS indicator is 4 mm long, a Type‐III mistake will cause the end of the active source to touch the inner end of the catheter channel. In most cases this will cause no issues from the TPS. Still, if a treatment plan based on the wrong measurement is delivered, the IDLs will obviously be shifted upstream by 2 mm (panel C of Fig. 7) with respect to the intended dose distribution (panel A of Fig. 7).

## 4 DISCUSSION

HDR brachytherapy is ideal for outpatient treatments, since it is more convenient than other inpatient procedures. The key request to physicists in a typical HDR brachytherapy procedure is to complete a timely plan while the patient is waiting for treatment; thus the ideal applicators should be easy to use and cause minimal chance of error. The applicator should be CT‐compatible so markers in each channel are visible, and transfer tubes should be applicator‐specific in the connector. Unfortunately, nonstandardized applicators meet none of these requirements.

For complicated, nonstandardized applicators such as the Miami Applicator for Tandem and Ovoid implants, special caution should be taken even if all restrictions in digitization mentioned above are followed. Under these conditions, a detail commissioning sheet for every channel and its corresponding transfer tube, as well as each total channel length, should be printed and at hand so as to avoid any mistake.

Furthermore, sometimes the FDPs of nonstandardized applicator channels are shorter than 1500 mm, even though standardized applicator‐specific tubes were used. For example, nonstandardized Miami applicator used for Tandem and Ovoid cases are presumably for 1500 mm FDPs. If this occurs, the delivery system would reject the plan because it is intended for treatment at longer FDPs. Therefore, the additional critical need is to have the treatment plan in place ahead of time whenever possible, since it will always be a major inconvenience to the patient if the treatment plan has to be corrected once the patient is set for treatment.

The detailed procedures on commissioning and digitizing nonstandardized applicators in this document are applicable to situations with standardized CT‐compatible applicators, if the markers are either missing or not properly inserted in the applicator channels during the CT scan. Still, we do recommend the CT should be taken with markers properly inserted into the applicators.

In this study, the standardized applicators for Nucletron remote afterloaders are used as references. The general approaches should be applicable to other remote afterloader systems, such as the VariSource™ afterloader of Varian Medical Systems, with a 1200 mm total channel length in its standardized GYN applicators.

We should also stress that standardized, flexible applicators of the same model may slightly differ in total channel length. It is therefore extremely important to commission each flexible applicator (or each channel of a multichannel applicator) before any patient treatment.

The major difference between the TPS of Nucletron and VariSource is that the Nucletron TPS simplifies a dwelling position into a point (or a spot), whereas the VariSource TPS displays the physical length of the HDR source. In the two systems, the dwell position definitions are remarkably different: the dwell position in a Nucletron system is the center of the source, but in a VariSource system the dwell position is located at the tip of the active wire. Nevertheless, the same principles presented in this study could be implemented in a VariSource system even though they were discussed here as applied to a Nucletron system.

## 5 CONCLUSIONS

We performed a comprehensive review and study on nonstandardized applicators for typical HDR procedures using Elekta Nucletron™ system, with a view to recommending strategies that overcome the dominant errors or uncertainties caused by incorrect digitization of the channel length and/or dwell positions. We considered situations that are likely to occur in HDR procedures, listed possible errors in each of them, and provided corresponding solutions.

## CONFLICT OF INTEREST

The authors declare no conflict of interest.
